# Supplementary material for: Crawling and Gliding: A Computational Model for Shape-Driven Cell Migration
Source: PLoS Comput Biol. 2015 Oct 21;11(10):e1004280. doi: 10.1371/journal.pcbi.1004280 (PMC4619082; doi:10.1371/journal.pcbi.1004280)
Supplement: S1 Code — (ZIP) [file pcbi.1004280.s012.zip › release/tst/doc/html/ca_8h_source.html]

Tissue Simulation Toolkit: ca.h Source File


Tissue Simulation Toolkit
 0.1.4.1


- Main Page
- Namespaces
- Classes
- Files

- File List
- File Members

ca.h

Go to the documentation of this file.

1 /\*

2

3 Copyright 1996-2006 Roeland Merks

4

5 This file is part of Tissue Simulation Toolkit.

6

7 Tissue Simulation Toolkit is free software; you can redistribute

8 it and/or modify it under the terms of the GNU General Public

9 License as published by the Free Software Foundation; either

10 version 2 of the License, or (at your option) any later version.

11

12 Tissue Simulation Toolkit is distributed in the hope that it will

13 be useful, but WITHOUT ANY WARRANTY; without even the implied

14 warranty of MERCHANTABILITY or FITNESS FOR A PARTICULAR PURPOSE.

15 See the GNU General Public License for more details.

16

17 You should have received a copy of the GNU General Public License

18 along with Tissue Simulation Toolkit; if not, write to the Free

19 Software Foundation, Inc., 51 Franklin St, Fifth Floor, Boston, MA

20 02110-1301 USA

21

22 \*/

23

24 // mainpage.h contains no C++ code, it is for the main page of the

25 // documentation

26 #include "mainpage.h"

27

29 #ifndef \_CA\_HH\_

30 #define \_CA\_HH\_

31 #include <vector>

32 #include <stdio.h>

33 #include "graph.h"

34 #include "pde.h"

35 //#include "dish.h"

36 #include "cell.h"

37

38 class Dish;

39

40 class Dir {

41

42  /\* To store a celldirection matrix \*/

43  friend class CellularPotts;

44 public:

45

46  Dir() {

47  aa1=0.; aa2=0.;

48  bb1=0.; bb2=0.;

49  lb1=0.; lb2=0.;

50  }

51

52  double aa1,aa2;

53  double bb1,bb2;

54  double lb1,lb2;

55 };

56

57 class CellularPotts {

58

59  friend class Info;

60  friend class Morphometry;

61

62 public:

64  CellularPotts(std::vector<Cell> \*cells, const int sizex=200,

65  const int sizey=200 );

66  // empty constructor

67  // (necessary for derivation)

68  CellularPotts(void);

69

70  // Keyword virtual means, that derived classed (cppvmCellularPotts) can override

71  // this function and carry out the memory allocation in their preferred way

72  // Every time AllocateSigma is called in the base class methods

73  // the function belonging the actual type will be called

74  virtual void AllocateSigma(int sx, int sy);

75

76  // destructor must also be virtual

77  virtual ~CellularPotts();

78

86  int \*\*SearchNandPlot(Graphics \*g=0, bool get\_neighbours=true);

87

89  inline void Plot(Graphics \*g) {

90  SearchNandPlot(g, false);

91  }

92

94  inline int \*\*SearchNeighbours(void) {

95  return SearchNandPlot(0, true);

96  }

97

99  inline int Mass(void) {

100  int mass=0;

101  for (int i=0;i<sizex\*sizey;i++) {

102  if (sigma[0][i]>0) mass++;

103  }

104  return mass;

105  }

106

111  void PlotSigma(Graphics \*g, int mag=2);

112

113

115  void DivideCells(void) {

116  std::vector<bool> tmp;

117  DivideCells(tmp);

118  }

119

128  void DivideCells(std::vector<bool> which\_cells);

129

133  int AmoebaeMove(PDE \*PDEfield=0);

134

139  void ReadZygotePicture(void);

140

141

142  // int BlobCounting(void); (not implemented?)

143

144  // Used internally to assign a Cell object to each "blob"

145  // "blobs" should already have different indices.

146  //

147  // (i.e. to start from a binary image you'd probably first want

148  // to apply a blob counting algorithm

149  void ConstructInitCells(Dish &beast);

150

152  inline int Time() const {

153  return thetime;

154  }

155

156

157  // not currently used? In Critter implementation (see Hogeweg

158  // 2000) this was used to have cells divide at double their original area.

159  inline int ZygoteArea() const {

160  return zygote\_area;

161  }

162

164  inline int SizeX() const {

165  return sizex;

166  }

167

169  inline int SizeY() const {

170  return sizey;

171  }

172

176  inline int Sigma(const int x, const int y) const {

177  return sigma[x][y];

178  }

179

180  // Was used to make it possible to enlarge the Graphics window in

181  // X11 and replace the contents interactively. Not currently supported.

182  void Replace(Graphics \*g);

183

189  Dir \*FindCellDirections(void) const;

190

193  int ThrowInCells(int n, int cellsize);

194

202  int GrowInCells(int n\_cells, int cellsize, double subfield=1.);

203  int GrowInCells(int n\_cells, int cell\_size, int sx, int sy, int offset\_x, int offset\_y);

204

206  inline Cell &AddCell(Dish &beast) {

207  cell->push\_back(Cell(beast));

208  return cell->back();

209  }

215  void ShowDirections(Graphics &g, const Dir \*celldir) const;

216

218  double MeanCellArea(void) const;

219

224  double CellDensity(void) const;

225

227  void ResetTargetLengths(void);

228

229  int spins\_converted;

230

236  void SetRandomTypes(void);

237

241  void GrowAndDivideCells(int growth\_rate);

242

243  inline Cell &getCell(int c) {

244  return (\*cell)[c];

245  }

246

250  double DrawConvexHull(Graphics \*g, int color=1);

251

256  double Compactness(double \*res\_compactness = 0,

257  double \*res\_area = 0,

258  double \*res\_cell\_area = 0);

259

260 private:

261  void IndexShuffle(void);

262  int DeltaH(int x,int y, int xp, int yp, PDE \*PDEfield=0);

263  bool Probability(int DH);

264  void ConvertSpin(int x,int y,int xp,int yp);

265  void SprayMedium(void);

266  int CopyvProb(int DH, double stiff);

267  void FreezeAmoebae(void);

268  void MeasureCellSizes(void);

269  void MeasureCellSize(Cell &c);

270  void CopyProb(double T);

271  bool ConnectivityPreservedP(int x, int y);

272

273  // little debugging function to print the site and its neighbourhood

274  inline void PrintSite(int x,int y) {

275  std::cerr << "--------\n";

276  std::cerr << "[" << sigma[x-1][y-1] << " " << sigma[x][y-1] << " " << sigma[x+1][y-1] << "]\n";

277  std::cerr << "[" << sigma[x-1][y] << " " << sigma[x][y] << " " << sigma[x+1][y] << "]\n";

278  std::cerr << "[" << sigma[x-1][y+1] << " " << sigma[x][y+1] << " " << sigma[x+1][y+1] << "]\n";

279  }

280

281 protected:

282  void BaseInitialisation(std::vector<Cell> \*cell);

283

284 protected:

285  int \*\*sigma;

286  int sizex;

287  int sizey;

288

289

290 private:

291  bool frozen;

292  static const int nx[25], ny[25];

293  static const int nbh\_level[5];

294  static int shuffleindex[9];

295  std::vector<Cell> \*cell;

296  int zygote\_area;

297  int thetime;

298  int n\_nb;

299 };

300

301

302 #endif

CellularPotts::sizey

int sizey

**Definition:** ca.h:287

CellularPotts::SizeY

int SizeY() const

Return the vertical size of the CA plane.

**Definition:** ca.h:169

CellularPotts::BaseInitialisation

void BaseInitialisation(std::vector< Cell > \*cell)

**Definition:** ca.cpp:72

pde.h

CellularPotts::Compactness

double Compactness(double \*res\_compactness=0, double \*res\_area=0, double \*res\_cell\_area=0)

**Definition:** ca.cpp:1380

Cell

**Definition:** cell.h:33

Dir::lb2

double lb2

**Definition:** ca.h:54

CellularPotts::SearchNandPlot

int \*\* SearchNandPlot(Graphics \*g=0, bool get\_neighbours=true)

Plots the dish to the screen or to a movie and searches the neighbours.

**Definition:** ca.cpp:475

CellularPotts::ReadZygotePicture

void ReadZygotePicture(void)

Read initial cell shape from XPM file. Reads the initial cell shape from an include xpm picture calle...

**Definition:** ca.cpp:600

Dir::aa1

double aa1

**Definition:** ca.h:52

CellularPotts::GrowInCells

int GrowInCells(int n\_cells, int cellsize, double subfield=1.)

Initialize the CA plane with n cells using an Eden growth algorithm.

**Definition:** ca.cpp:1075

CellularPotts::sigma

int \*\* sigma

**Definition:** ca.h:285

CellularPotts::Morphometry

friend class Morphometry

**Definition:** ca.h:60

CellularPotts::ConstructInitCells

void ConstructInitCells(Dish &beast)

**Definition:** ca.cpp:664

CellularPotts::PlotSigma

void PlotSigma(Graphics \*g, int mag=2)

**Definition:** ca.cpp:464

CellularPotts::DivideCells

void DivideCells(void)

Divide all cells.

**Definition:** ca.h:115

CellularPotts::AllocateSigma

virtual void AllocateSigma(int sx, int sy)

**Definition:** ca.cpp:148

CellularPotts::getCell

Cell & getCell(int c)

**Definition:** ca.h:243

CellularPotts::Plot

void Plot(Graphics \*g)

Plot the dish to Graphics window g.

**Definition:** ca.h:89

CellularPotts::SetRandomTypes

void SetRandomTypes(void)

Give each cell a random cell type.

**Definition:** ca.cpp:1278

CellularPotts

**Definition:** ca.h:57

CellularPotts::ShowDirections

void ShowDirections(Graphics &g, const Dir \*celldir) const

Display the division planes returned by FindCellDirections.

**Definition:** ca.cpp:894

CellularPotts::sizex

int sizex

**Definition:** ca.h:286

CellularPotts::Replace

void Replace(Graphics \*g)

cell.h

CellularPotts::FindCellDirections

Dir \* FindCellDirections(void) const

**Definition:** ca.cpp:765

PDE

**Definition:** pde.h:31

Dir::bb1

double bb1

**Definition:** ca.h:53

Dish

The virtual Petri dish.

**Definition:** dish.h:43

CellularPotts::ZygoteArea

int ZygoteArea() const

**Definition:** ca.h:159

CellularPotts::~CellularPotts

virtual ~CellularPotts()

**Definition:** ca.cpp:140

Dir::bb2

double bb2

**Definition:** ca.h:53

Dir::Dir

Dir()

**Definition:** ca.h:46

Dir::lb1

double lb1

**Definition:** ca.h:54

CellularPotts::MeanCellArea

double MeanCellArea(void) const

Returns the mean area of the cells.

**Definition:** ca.cpp:1246

CellularPotts::GrowAndDivideCells

void GrowAndDivideCells(int growth\_rate)

**Definition:** ca.cpp:1295

CellularPotts::AmoebaeMove

int AmoebaeMove(PDE \*PDEfield=0)

Monte Carlo Step. Returns summed energy change.

**Definition:** ca.cpp:375

CellularPotts::Time

int Time() const

Returns the number of completed Monte Carlo steps.

**Definition:** ca.h:152

graph.h

CellularPotts::Mass

int Mass(void)

Return the total area occupied by the cells.

**Definition:** ca.h:99

CellularPotts::SearchNeighbours

int \*\* SearchNeighbours(void)

Searches the cells' neighbors without plotting.

**Definition:** ca.h:94

CellularPotts::spins\_converted

int spins\_converted

**Definition:** ca.h:229

Dir

**Definition:** ca.h:40

CellularPotts::ThrowInCells

int ThrowInCells(int n, int cellsize)

Initialize the CA plane with n circular cells fitting in a cellsize^2 square.

**Definition:** ca.cpp:1011

Graphics

API for Graphics windows.

**Definition:** graph.h:36

CellularPotts::AddCell

Cell & AddCell(Dish &beast)

Adds a new Cell and returns a reference to it.

**Definition:** ca.h:206

CellularPotts::SizeX

int SizeX() const

Return the horizontal size of the CA plane.

**Definition:** ca.h:164

mainpage.h

CellularPotts::CellDensity

double CellDensity(void) const

Returns the cell density.

**Definition:** ca.cpp:1233

Dir::aa2

double aa2

**Definition:** ca.h:52

CellularPotts::CellularPotts

CellularPotts(void)

**Definition:** ca.cpp:114

CellularPotts::ResetTargetLengths

void ResetTargetLengths(void)

Set target lengths of all cells to the value given in parameter file.

**Definition:** ca.cpp:1265

Info

Enables interactive querying of the simulation.

**Definition:** info.h:36

CellularPotts::DrawConvexHull

double DrawConvexHull(Graphics \*g, int color=1)

**Definition:** ca.cpp:1328

CellularPotts::Sigma

int Sigma(const int x, const int y) const

Return the value of lattice site (x,y).

**Definition:** ca.h:176


---

Generated on Thu Aug 14 2014 22:04:01 for Tissue Simulation Toolkit by  

 1.8.6
